# Monoamine and genome-wide DNA methylation investigation in behavioral addiction

**DOI:** 10.1038/s41598-020-68741-5

**Published:** 2020-07-16

**Authors:** Yui Asaoka, Moojun Won, Tomonari Morita, Emi Ishikawa, Young-A Lee, Yukiori Goto

**Affiliations:** 1 Primate Research Institute, Kyoto University, Inuyama, Aichi 484-8506 Japan; 2 Kyowa Hospital, Obu, Aichi 474-0071 Japan; 3 Department of Food Science and Nutrition, Daegu Catholic University, Gyeongsan, Gyeongbuk 38430 South Korea

**Keywords:** Addiction, Human behaviour, Epigenetics and behaviour

## Abstract

Behavioral addiction (BA) is characterized by repeated, impulsive and compulsive seeking of specific behaviors, even with consequent negative outcomes. In drug addiction, alterations in biological mechanisms, such as monoamines and epigenetic processes, have been suggested, whereas whether such mechanisms are also altered in BA remains unknown. In this preliminary study with a small sample size, we investigated monoamine concentrations and genome-wide DNA methylation in blood samples from BA patients and control (CT) subjects. Higher dopamine (DA) metabolites and the ratio between DA and its metabolites were observed in the BA group than in the CT group, suggesting increased DA turnover in BA. In the methylation assay, 186 hyper- or hypomethylated CpGs were identified in the BA group compared to the CT group, of which 64 CpGs were further identified to correlate with methylation status in brain tissues with database search. Genes identified with hyper- or hypomethylation were not directly associated with DA transmission, but with cell membrane trafficking and the immune system. Some of the genes were also associated with psychiatric disorders, such as drug addiction, schizophrenia, and autism spectrum disorder. These results suggest that BA may involve alterations in epigenetic regulation of the genes associated with synaptic transmission, including that of monoamines, and neurodevelopment.

## Introduction

Behavioral addiction (BA) is a psychiatric condition characterized by repeated, impulsive and compulsive seeking of specific behavioral processes, even though consequent negative outcomes^1–3^. In the current diagnostic manuals, such as the DSM-5^4^ and ICD-11^5^, pathological gambling and Internet and gaming disorders are officially categorized into this psychiatric disorder. However, in many preclinical studies and the realms of clinical setting, impulse control disorders, such as kleptomania (KM), paraphilic (PP) and nonparaphilic sexual disorder, are also thought to meet the criteria of BA, and are, therefore, quite often considered BA^6^.

BA and drug addictions are known to be comorbid with several psychiatric disorders, such as schizophrenia (SCZ) and autism spectrum disorder (ASD). It has long been known that drug addiction is highly prevalent in SCZ^7,8^. Moreover, BA such as pathological gambling is also higher in SCZ patients^9,10^. Studies have shown associations of drug addiction and BA with ASD, with a higher prevalence of drug addiction and BA, such as gaming disorder, in individuals with ASD^11–14^. In particular, a recent study suggests that common molecules and pathways may be involved between drug addiction and ASD^15^.

Since BA is still a conceptually new disorder, it has remained largely unclear to what extent BA may share its biological mechanisms with those of drug addiction. One of the central mechanisms involved in drug addiction is indeed monoamine transmission, particularly dopamine (DA)^16,17^, and less suggested for norepinephrine (NE)^18^ and serotonin (5-HT)^19^, given that most, if not all, addictive substances stimulate the DA system. Animal model studies have demonstrated that some addictive drugs, such as amphetamine and cocaine, have been shown to decrease the basal, and tonic DA levels in the striatum^20,21^, which, in turn, could increase phasic DA release upon taking these drugs^22^. Nevertheless, only a few studies have examined whether monoamine concentrations may be altered in peripheral blood samples in addicted patients. A study has reported that plasma DA and one of the metabolites of norepinephrine (NE), 3-methoxy-4-hydroxyphenylglycol (MHPG), were increased with the duration of heroin and cocaine use in patients^23^, whereas another study has shown decreased plasma levels of the DA metabolite homovanillic acid (HVA) in individuals with alcohol addiction depending on the genetic backgrounds of catechol-o-methyltransferase (COMT)^24^. In relation to BA, an association of blood DA level and Internet addiction in adolescents has been demonstrated^25^.

Accumulating evidence suggests that epigenetic processes also play pivotal roles in drug addiction, whereas this has not yet been examined in BA. Exposures to addictive substances could alter gene expression in a tissue-specific manner in the brain through epigenetic processes^26^; alternatively, epigenetic changes could occur early in development, predisposing an individual to increased vulnerability to addiction^27^. Indeed, similar processes are thought to take place in BA, which is essentially initiated by environmental stimuli in adulthood or during development. Recent studies with genome-wide DNA methylation assays have unveiled alterations in methylation patterns in drug addiction patients compared with healthy subjects^28,29^. DNA methylation is an epigenetic process that regulates gene expression, and as such, it was thought that CpG sites on promoter regions play crucial roles in inhibiting gene expression^30^. However, more recent studies have unveiled that CpG sites are also substantially located in other regions, including gene bodies, and the methylation of such CpGs in gene bodies is also involved in the regulation of gene expression^31–33^.

Collectively, in this study, we investigated whether monoamine concentrations were altered in BA patients, and whether such alterations were associated with epigenetic processes, such as DNA methylation. To address this issue, blood samples were obtained from BA patients and control (CT) subjects, and a high-performance liquid chromatography (HPLC) assay was conducted to measure monoamine concentrations. Moreover, a genome-wide DNA methylation assay with an Infinium HumanMethylationEPIC BeadChip array was conducted to compare the methylation status of DNA between BA patients and CT subjects, particularly whether DNA methylation on any genes associated with monoamine transmission was altered.

## Results

### Subjects

Blood samples were obtained from 24 healthy CT adults and from hospitalized patients who were diagnosed with BA (n = 16), who were further divided into patients with symptoms of gambling (n = 1), kleptomania (KM; n = 10) and paraphilia (PP; n = 5). All samples of 24 CT subjects and 16 BA patients were processed for blood monoamine assay with HPLC, whereas the genome-wide DNA methylation assay was conducted with samples from 10 CT subjects and 5 each from KM and PP patients, totaling 10 BA patients. Ten CT and 5 KM samples for the methylation assay were selected from the entire subjects based on characteristics of symptoms, for which the attending physician described as most typical, along with the consideration of matching for age and sex.

### Blood monoamine concentrations

**Figure 1:**
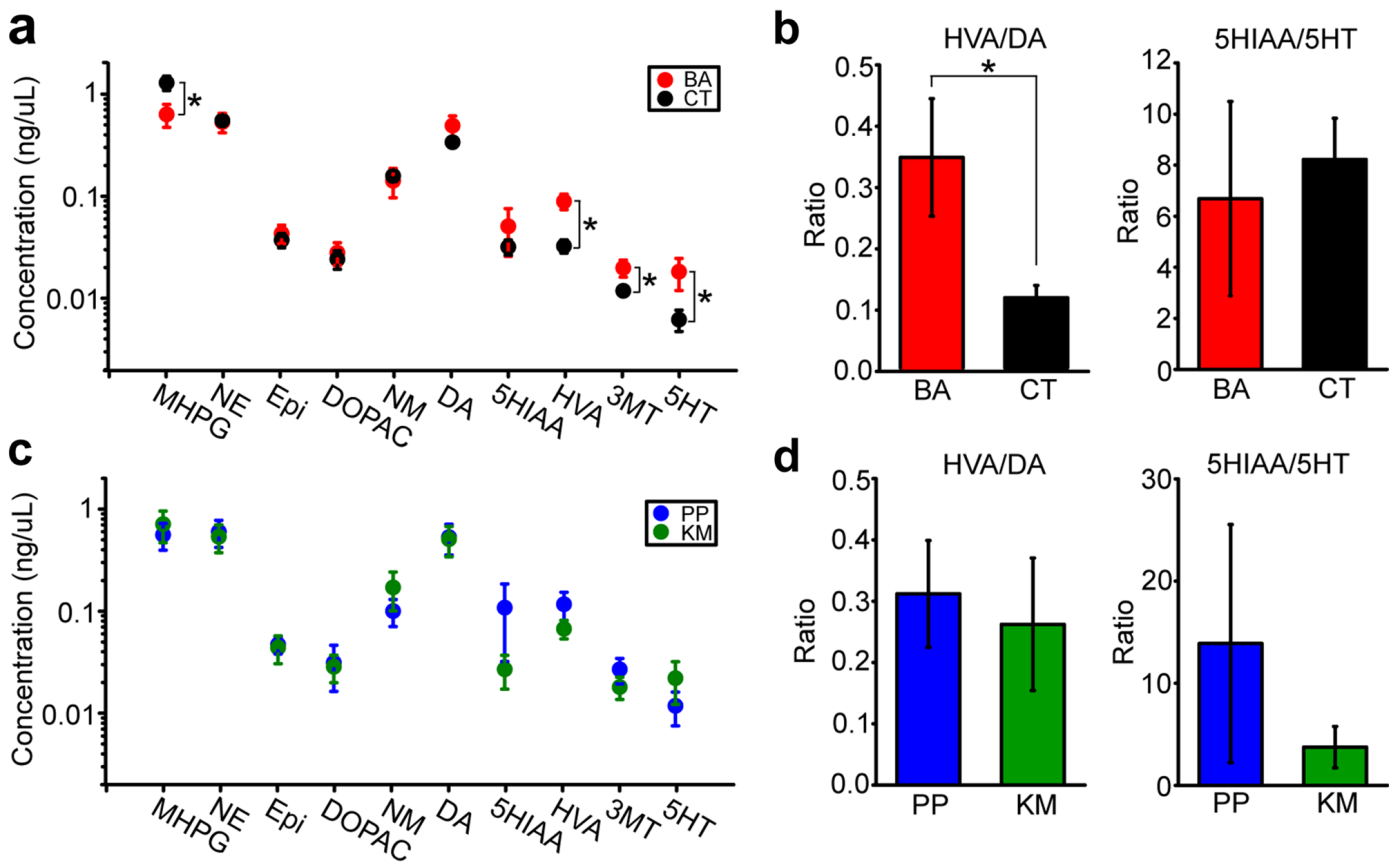
Blood monoamine concentrations in BA patients and CT subjects. (**a**) A graph showing comparisons of monoamine concentrations between BA patients and CT subjects. *DA* dopamine, *5-HT* serotonin, *NE* norepinephrine, *Epi* epinephrine, *MHPG* 3-methoxy-4-hydroxyphenylglycol, *NM* normetanephrine, *DOPAC* 3,4-dihydroxyphenylacetic acid, *HVA* homovanillic acid, *3-MT* 3-methoxytyramine, *5-HIAA* 5-hydroxyindole acetic acid. Error bars indicate the s.e.m. *p < 0.05. (**b**) Bar graphs showing the ratio of one of the DA metabolites, HVA and DA (left) and that of the 5-HT metabolite, 5-HIAA and 5-HT (right), in BA patients and CT subjects. (**c**,**d**) Graphs similar to (**a**) and (**b**) but showing comparisons between KM and PP patients.

HPLC assays were conducted to measure the blood concentrations of DA, 5-HT, NE, and epinephrine (Epi) and their metabolites, MHPG, normetanephrine (NM), 3,4-dihydroxyphenylacetic acid (DOPAC), HVA, 3-methoxytyramine (3-MT), and 5-hydroxyindole acetic acid (5-HIAA). MHPG was significantly lower (unpaired *t* test, t_38_ =  − 2.30, p = 0.027), whereas HVA (t_38_ = 4.08, p < 0.001), 3-MT (t_38_ = 2.33, p = 0.025), and 5-HT (t_38_ = 2.22, p = 0.032) were significantly higher, in blood samples from BA patients than in those from CT subjects (Fig. 1a). Moreover, the ratio of one DA metabolite, HVA, to DA was significantly higher in BA patients than in CT subjects (t_38_ = 2.80, p = 0.008), whereas the ratio of the 5-HT metabolite 5-HIAA, to 5-HT was not different between BA patients and CT subjects (Fig. 1b). Monoamine concentrations of BA patients were further analyzed separately for KM and PP patients. However, none of the measurements were significantly different between KM and PP patients (Fig. 1c,d). These results suggest that increased DA turnover may be involved in BA regardless of its symptom types.

### Genome-wide DNA methylation assay

**Figure 2:**
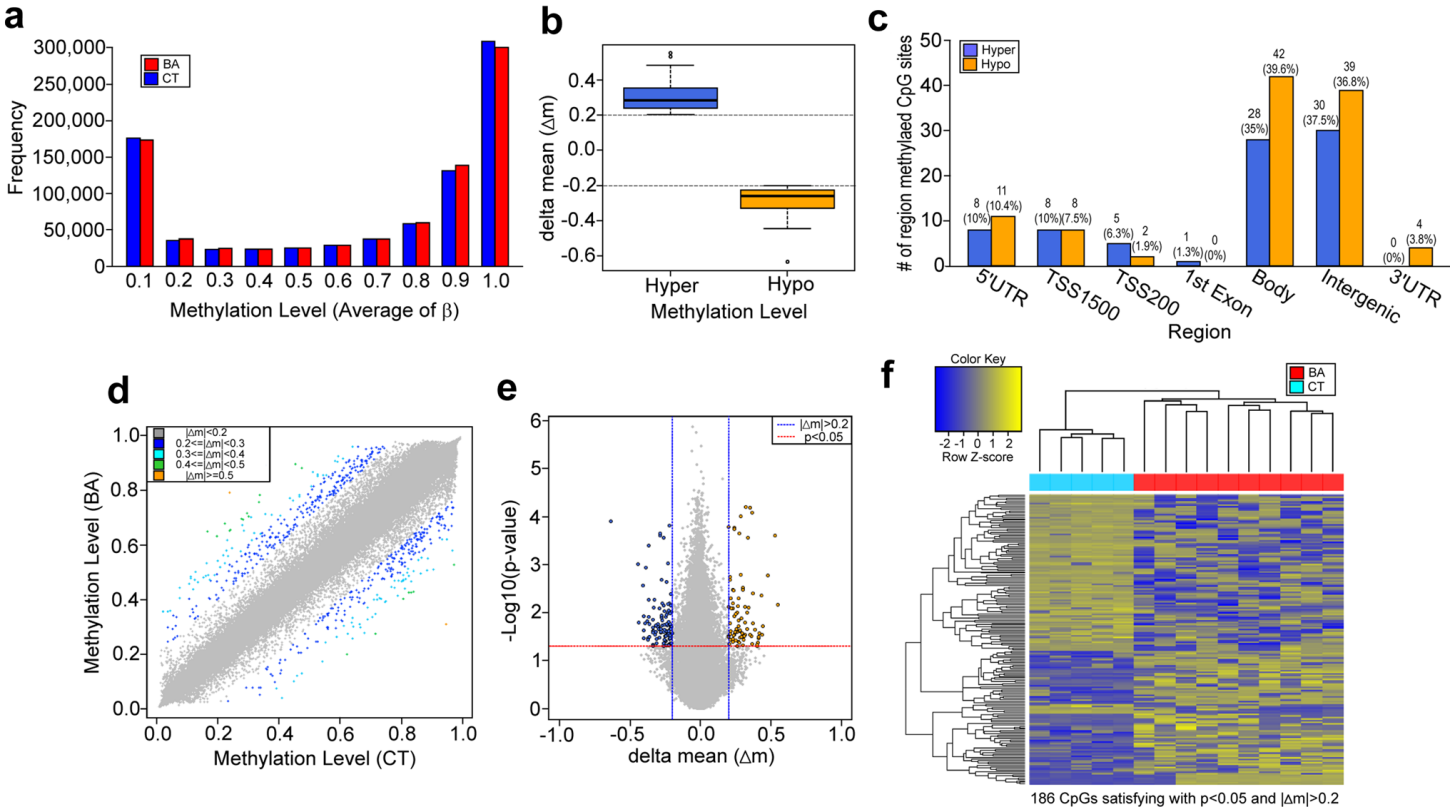
Genome-wide DNA methylation assay in BA patients and CT subjects. (**a**) A histogram showing the distribution of methylation status on CpGs in BA patients and CT subjects. (**b**) A box plot showing the levels of hyper- and hypomethylated CpGs in BA patients compared to CT subjects. The dashed line indicates the cut-off (|∆mean| > 0.2) for hyper- and hypo-methylation. (**c**) A bar graph showing the distribution of functional locations for hyper- and hypomethylated CpGs. (**d**) A scatter plot showing methylation status of the BA group versus the CT group, with the color coding for the level of hypo- and hypermethylation comparing between the BA group and the CT group. (**e**) A volcano plot for ∆mean versus p-value in *t* test, with the color illustrating the hyper- and hypomethylation that fill the criteria of |∆mean| ≥ 0.2 and p < 0.05. (**f**) A heat map showing hierarchical clustering on distance similarity for samples and CpGs between the BA group and the CT group.

Genome-wide DNA methylation in DNA extracted from blood samples of BA patients and CT subjects was examined with an Infinium HumanMethylationEPIC BeadChip array. The ratio of methylated probe intensity to the overall intensity (β-value) of 845,978 CpG sites after data preprocessing and quality checks was distributed more below 0.1 or above 0.9 (Fig. 2a), suggesting that many CpG sites were either unmethylated or fully methylated in both the BA group and CT group. Among these CpGs, 106 hypomethylated (∆mean ≤  − 0.2, where ∆mean = mean(average of β in BA group) − mean(average of β in CT group), and p < 0.05 in unpaired *t* test) and 80 hypermethylated (∆mean ≥ 0.2) CpGs, with odds ratios varying from 0.015 to 58.6 and fold changes varying from − 8.51 to 11.4, respectively, were identified in the BA group compared to the CT group (Fig. 2b,d,e; Suppl. Data S1). However, these methylation differences were modest at most, and none of them was significant with multiple testing correction using the Benjamini–Hochberg procedure^34^ (Suppl. Data S1, fdr.p.val). Many of these 186 CpG sites were located in the gene bodies and intergenic regions (Fig. 2c). A heat map of hierarchical clustering on distance similarity for samples and CpGs (Euclidean distance, complete linkage), using the M-value (the log2 ratio of the intensities of methylated probe versus unmethylated probe) of significant data, shows distinct methylation patterns between the BA group and the CT group (Fig. 2f).

### Functional associations and relevance to other psychiatric disorders

Given that the methylation status of DNA in peripheral blood tissues is not necessarily identical to that in brain tissues^35^, we further analyzed whether there was correspondence in methylation level between the blood and brain tissues for 186 identified CpGs with hyper- and hypomethylation, using the database at IMAGE-CpG^35^. With this analysis, 64 CpGs with hyper- and hypomethylation were identified (Suppl. Data S2), and subsequent analyses were conducted with the genes identified where those 64 CpGs are present.

**Figure 3:**
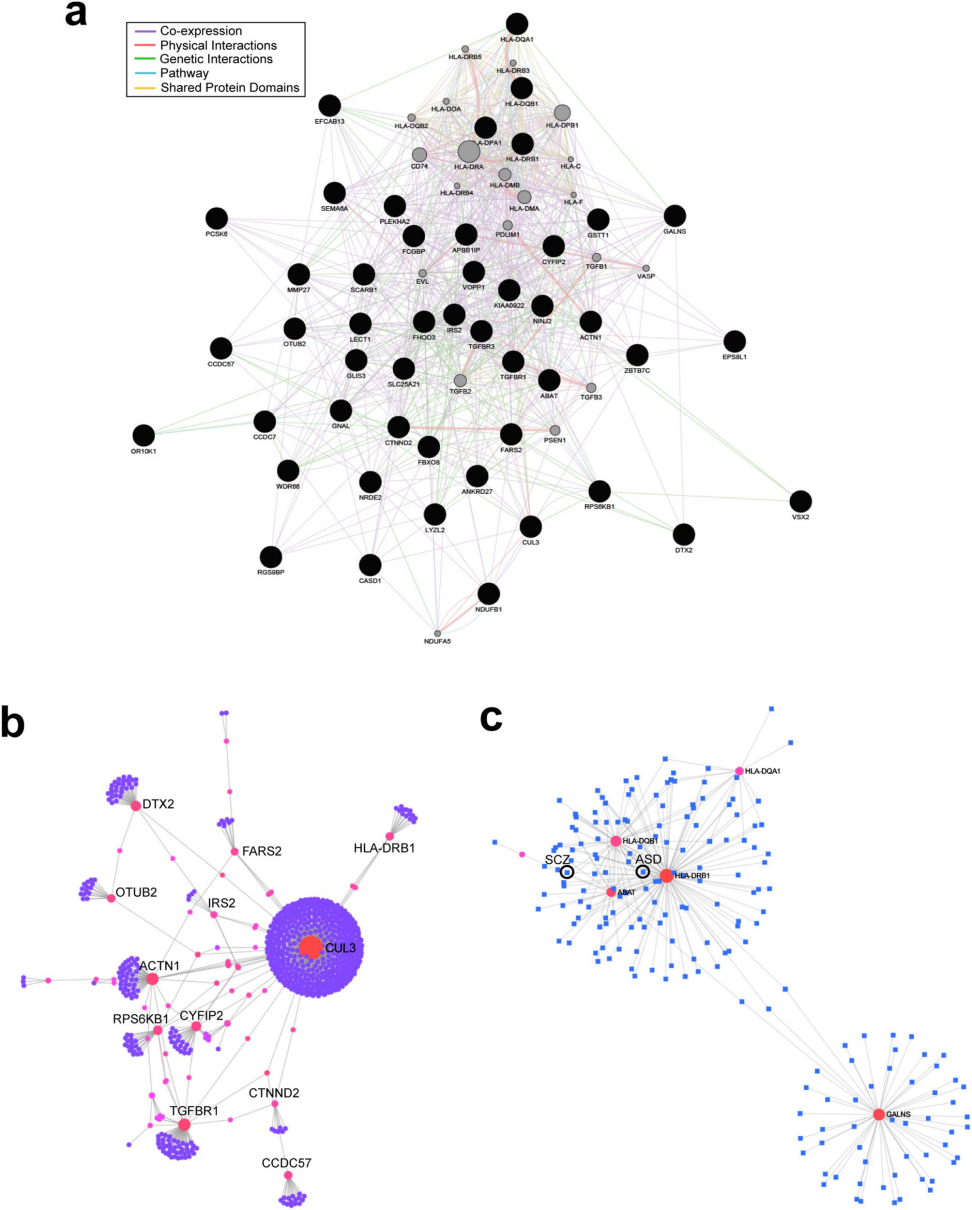
Gene network analyses for the genes with hyper- and hypomethylation in BA patients. (**a**) A diagram illustrating gene network analysis in which interactions of the genes (coexpression, physical interactions, genetic interactions, pathway, and shared protein domains) are shown. (**b**) A diagram similar to (**a**) but showing network analysis for protein–protein interactions in the nucleus accumbens constructed with the genes with identified hyper- and hypomethylation. (**c**) A network diagram similar to (**a**) but showing network analysis for gene–disease associations.

First, gene network analysis was conducted using the database at GeneMANIA^36^. This analysis unveiled extensive associations among the identified genes (coexpression, 91.3%; physical interactions, 5.92%; genetic interactions, 1.46%; pathway, 0.96%; shared protein domains, 0.33%; Fig. 3a), along with significant convergence (Q-value < 0.1) identifying 118 gene ontologies, which were primarily associated with membrane trafficking, such as transport vesicle and endocytic vesicle membrane, and immune functions, such as MHC class II receptor activity and cellular response to interferon-gamma (Suppl. Data S3).

Further analyses were conducted using the database at NetworkAnalyst^37^. An example of tissue specific protein–protein interactions in the nucleus accumbens is illustrated in Fig. 3b, which demonstrates CUL3 as the largest node, along with several other nodes, such as ACTN1, DTX2, and TGFBR1 (Suppl. Data S4). Similar network structures with these genes as nodes also emerged for the frontal cortex, hippocampus, amygdala, and substantia nigra.

Gene–disease associations were further evaluated with network analysis (Fig. 3c; Suppl. Data S5). This analysis unveiled associations of the identified genes with psychiatric disorders, such as ASD (degree of convergence = 2; betweenness = 1,388) and SCZ (degree of convergence = 2; betweenness = 31.51). In addition, more detailed gene–disease associations were also examined using the database at DisGeNET^38^, with which 37 out of 64 genes were identified for associations with diseases, including psychiatric disorders (Suppl. Data S6). Among such psychiatric disorders, the strongest associations were observed for intellectual disability (9 genes; CTNND2, GNAL, VSX2, TGFBR1, CUL3, FBXO8, GALNS, ABAT, GLIS3), ASD (8 genes; HLA-DRB1, HLA-DQB1, CTNND2, IRS2, HLA-DRB1, ABAT, CTNND2, PCSK6) and SCZ (8 genes; HLA-DRB1, HLA-DQB1, GSTT1, GNAL, CTNND2, CUL3, HLA-DQA1, GLIS3). In addition, SEMA6A, GSTT1, and SCARB1, which are included in the identified 64 genes with hyper- and hypomethylation, were associated with drug addiction (alcohol, amphetamine, cannabis, cocaine, marijuana, and phencyclidine).

These results suggest that the genes in which hyper- and hypomethylation were observed in the BA group may be associated with biological functions such as membrane trafficking, which may affect synaptic transmission in the brain, and the immune system. Moreover, some of these genes associated with BA may overlap with candidate genes of other psychiatric disorders, such as SCZ and ASD. In particular, SEMA6A, GSTT1, and SCARB1 could be candidate genes that may be mutually involved in BA and drug addiction.

### DNA methylation differences between KM and PP

**Figure 4:**
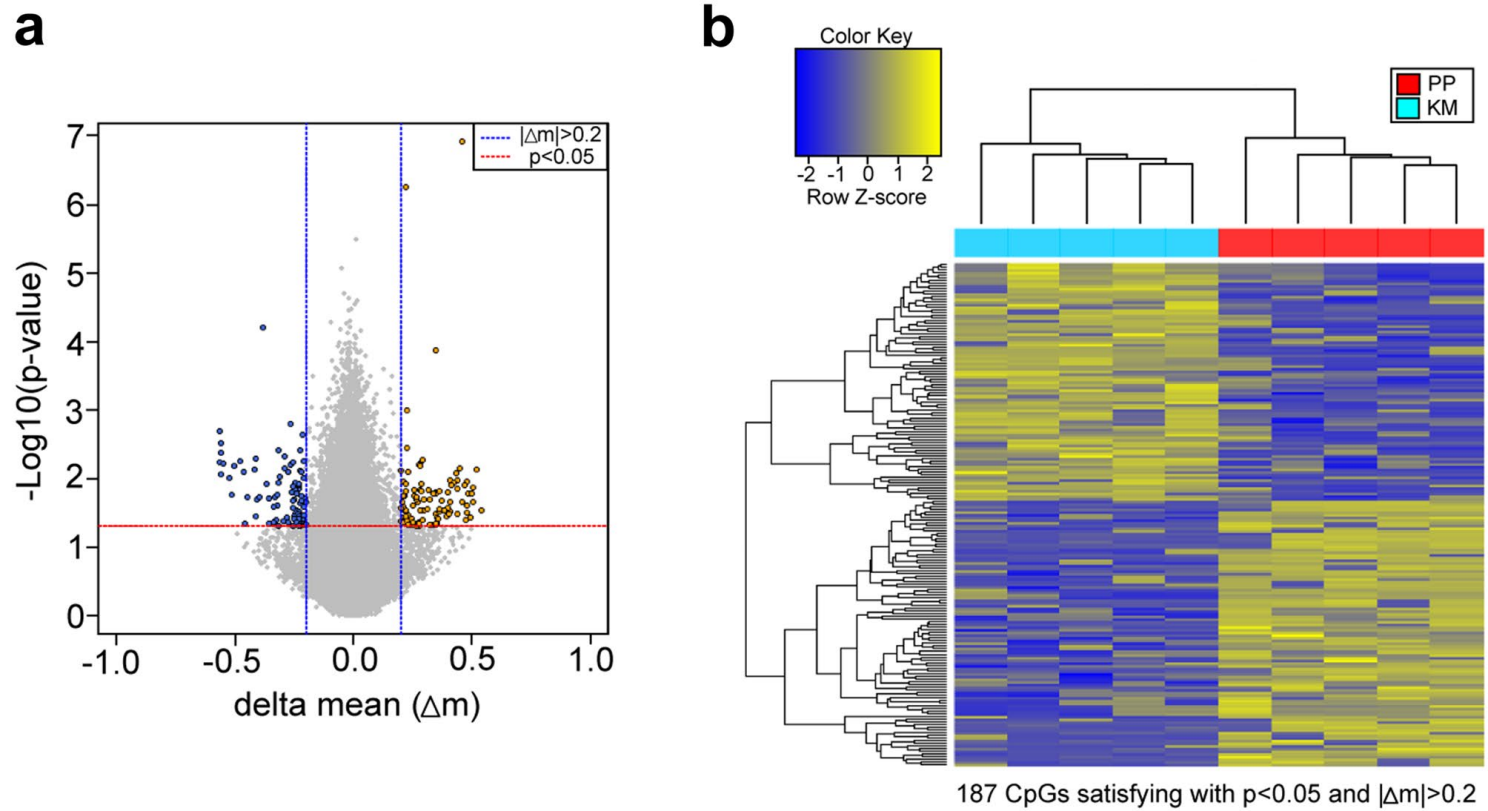
Comparisons of genome-wide DNA methylation assays between KM and PP patients. (**a**) A volcano plot for ∆mean versus p-value in the *t* test, showing hyper- and hypomethylation in KM patients compared to PP patients. (**b**) A heat map showing hierarchical clustering on distance similarity for samples and CpGs between the KM group and the PP group.

Since the BA group manifested heterogeneous symptoms, we analyzed genome-wide DNA methylation data separately for patients with KM and PP. When the KM and PP groups were compared, 88 hypo- and 99 hypermethylated CpG sites were identified in the KM group compared to the PP group (Fig. 4a; Suppl. Data S7). A heat map of hierarchical clustering also showed distinct methylation patterns between the KM group and the PP group (Fig. 4b). These results suggest that methylation patterns may be different between KM and PP, although differences in other measurements, such as blood monoamine concentrations, are less clear between them.

### Receiver operating characteristic analyses of monoamines and methylation

One of the important implications of the data is that DNA methylation and monoamines might be developed into biomarkers. The fact that it is possible to observe DNA methylation and monoamine concentration changes in blood suggests feasibility. Thus, we conducted receiver operator characteristic (ROC) analyses^39^ to examine the sensitivity and specificity of these markers.

**Figure 5:**
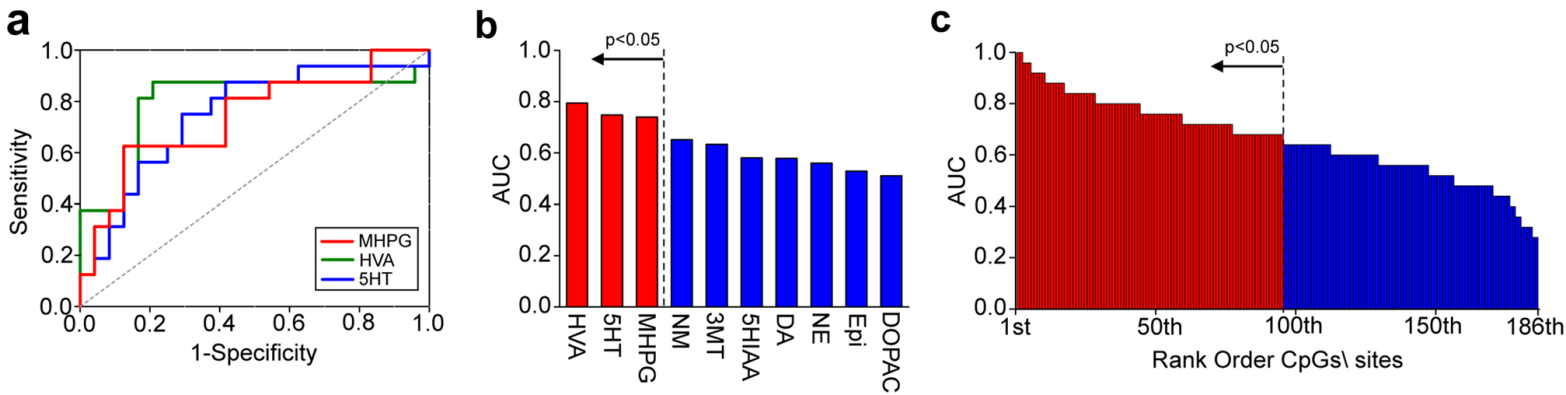
ROC analyses of monoamines and DNA methylation. (**a**) A graph showing ROC curves of MHPG, HVA, and 5-HT, which are significantly higher or lower in BA patients than in CT subjects. (**b**) A bar graph showing the AUCs of all monoamines, with red and blue bars representing monoamines whose AUCs are higher or lower than the asymptotic p-value of 0.05, respectively. (**c**) A graph similar to (**b**) but showing AUCs of 186 hyper- or hypomethylated CpGs in BA patients. These 186 CpGs are ranked from the 1st to 186th based on the values of AUCs.

Although the reliability of analyses was limited by the small sample sizes, ROC analyses revealed that blood concentrations of HVA, 5-HT, and MHPG, which were significantly lower or higher in BA patients than in CT subjects, successfully distinguished BA patients from CT subjects (the area under the curve (AUC) significantly larger than 0.5 with asymptotic p-value < 0.05), but other monoamines did not (Fig. 5a,b). A similar ROC analysis was also conducted with 186 hyper- or hypomethylated CpG sites. AUCs with asymptotic p-values < 0.05 were observed in 95 out of these 186 hyper- or hypomethylation sites (Fig. 5c; Suppl. Data S8).

These results suggest that some blood monoamine concentrations and blood cell DNA methylation status could distinguish BA and CT, and thereby might be used as biomarkers of BA.

## Discussion

In this preliminary study with a small sample size, we have shown that increased DA turnover and DNA methylation differences are involved in BA. Such DNA methylation differences appear to occur in genes that play roles in cell membrane trafficking and the immune system and are associated with other psychiatric disorders, such as intellectual disability, ASD, SCZ, and drug addiction. Moreover, although DA turnover was not different, DNA methylation was clearly distinct between the symptoms of KM and PP. In particular, none of participants who were subjected to investigation in either the BA group or the CT group had any specific chronic diseases or were on medication, excluding the possibility of such effects on alterations in blood monoamine concentrations and DNA methylation.

There are several limitations in this study, among which the most obvious and important one is the small sample size, with n = 10 for BA patients, and n = 5 for control subjects. In addition, BA patients were further divided into KM (n = 5) and PP (n = 5) patients. Thus, the results reported in this study must be interpreted cautiously under such limitations. This is particularly important, since all 186 CpGs with statistically significant hyper- or hypomethylation identified in BA patients compared to those of controls were not robust, and none of them reached statistically significant differences with multiple testing correction using the Benjamini–Hochberg procedure. Nevertheless, it is still reasonable that functional analysis is performed on a larger set of genes including only nominally significant sites to explore whether important functional interrelationships exist. Further analysis with a larger sample number is crucial and may still potentially yield a different picture of epigenetic alterations in BA. Another limitation is that no validation was conducted on the results by a different method, such as pyrosequencing or targeted sequencing, to confirm hyper- and hypomethylation in BA patients. Indeed, since this study included only a small number of samples, with only modestly significant sites, the validation of such significant sites is important. Although we did not conduct such post-hoc validation by a different sequencing method, reproducibility between samples using the M value with 1,000 randomly selected CpGs was very high (Pearson’s correlation coefficient of 0.98 or higher in all paired cases; Suppl. Fig. S1). Moreover, a recent study by Noble and colleagues demonstrated that the detection of methylation level changes with the Illumina Infinium Methylation EPIC BeadChip and bisulfite-based amplicon sequencing (BSAS) in subjects with cannabis and tobacco users versus nonusers is highly correlated (adjusted R^2^ = 0.8878 and 0.8683 in BSAS vs. EPIC and vice versa, respectively)^40^. Such correlations are found to be even more accurate at modest levels of methylation changes than robust ones.

In this study, we examined blood monoamine concentrations, given previous studies showing correlations between blood and brain monoamine levels. For instance, in human subjects, plasma concentrations of the DA metabolite, HVA, were found to be increased with stress^41^ and physical activity^42^, which is consistent with an increase in DA release by stress^43^ and physical activity^44^ in the striatum. The 2-deoxyglucose (2DG)-induced increase in DA in the striatum has also been indirectly correlated with increased plasma HVA in human subjects^45,46^. In animal studies, the stimulation of the nigrostriatal pathway increases, whereas lesions of the pathway decrease plasma DA metabolites^47^. The effects of D2 antagonists and apomorphine on the brain have also been demonstrated to alter plasma HVA concentrations^48^. In our study, a higher concentration of HVA, but not of DA itself, was observed, along with a higher HVA/DA ratio, in BA patients than in CT subjects. Collectively, these observations suggest that DA release may be increased in the brains of BA patients. In relation to this finding, other studies have shown an association between increased blood DA and HVA concentrations and Internet addiction^25^ and alcoholism^24^, although in Internet addiction, DA is elevated, and whether its metabolites are increased remains unknown. In alcoholism, a genetic background of COMT appears to play a significant role in causing such increased plasma HVA concentrations. In addition to DA, a decrease in one NE metabolite, MHPG, and an increase in 5-HT, but not its metabolite, were observed in BA patients. Although both NE^18^ and 5-HT^19^ transmission have been suggested in drug addiction, how such lower MHPG and higher 5-HT levels may be involved in BA remains less clear.

Given that epigenetic processes have been suggested in the mechanisms of drug addiction^49,50^, an analysis of genome-wide DNA methylation was conducted to examine whether underlying epigenetic differences might exist between BA patients and CT subjects. This analysis unveiled 186 hyper- or hypomethylated CpG sites in BA patients compared to CT subjects, most of which were located on gene bodies and intergenic regions but not in promoter regions. Although such methylation on gene bodies has been relatively unexplored, accumulating evidence suggests that methylation in gene bodies is also crucial in the regulation of gene expression^31–33^. Approximately one third of CpG sites were found to be correlated with the methylation status of DNA in brain tissues with the IMAGE-CpG database, many of which were, in fact, on the gene bodies. Thus, we conducted gene network analysis to examine the biological functions of the genes in which hyper- or hypomethylated CpGs are present. No direct association was found for monoamine transmission. Instead, this network analysis revealed that genes with hyper- and hypomethylation in BA are involved in cell membrane trafficking and the immune system. Indeed, cell membrane trafficking is the major machinery of synaptic release^51,52^. Accumulating evidence also suggests that cell membrane trafficking plays important roles in neurodevelopment^53^. The identified immune pathways or genes include major histocompatibility complex (MHC)-class II, T cells, and Interferon-gamma (IFNγ). Studies have shown that MHC-class I is involved in neuronal functions, such as synaptic release, axonal regeneration, synaptic plasticity and neurodevelopment^54,55^. Moreover, MHC-class I is expressed in dopamine neurons, which, in turn, suppress relapse to reward seeking^56^. In contrast, the expression of MHC-class II in the central nervous system is found only in glial cells, such that its roles are more limited than MHC-class I^57^. Recent studies have demonstrated that T cells yield substantial influence on cognitive functions and the underlying neural activities^58^. In particular, consistent with our finding, a study has now shown that the T cell DNA methylation profile is different in individuals with drug addiction than that in healthy subjects^59^. IFNγ is a cytokine whose role in neuronal functions and behaviors such as social interactions has also been reported^60,61^.

Tissue specific protein–protein interaction analysis was conducted with the online database based on the genes identified with hyper- and hypomethylation. A similar protein–protein interaction network emerged regardless of brain areas, including the frontal cortex, basal ganglia, nucleus accumbens, hippocampus, amygdala, and substantia nigra. Hubs of proteins in this network included CUL3, ACTN1, DTX2, and TGFBR1. CUL3 was the largest node in the network, whose deficiency has been shown to cause social deficits and heightened anxiety^62^. ACTN1 is a postsynaptic scaffolding protein that interacts with two other postsynaptic scaffolding proteins, SHANK3 and HOMER3^63^. The Homer family of proteins, including HOMER3, is suggested to moderate neuroplasticity associated with drug addiction^64^. In addition, the downregulation of DTX expression with chronic intermittent ethanol exposure^65^, and the upregulation of TGFBR1 with withdrawal from cocaine self-administration in the nucleus accumbens^66^ have been reported in rodent studies.

It is of particular interest to note that gene–disease association analysis also unveiled associations of the gene with hyper- and hypomethylation in BA patients with intellectual disability, SCZ, and ASD. Indeed, deficits in synaptic transmission and neurodevelopment are suggested in intellectual disability, SCZ, and ASD^67–70^. In addition, some of the genes, such as SEMA6A, GSTT1, and SCARB1 are also associated with addiction to an assortment of drugs, including alcohol, amphetamine, cannabis, cocaine, marijuana, and phencyclidine, and, therefore, could be candidate genes that may be mutually involved in BA and drug addiction.

In conclusion, our study suggests that peripheral blood DNA methylation status and monoamine concentrations may be altered in BA. The genes identified with hyper- or hypomethylation in BA may play roles in cell membrane trafficking and immune functions. Moreover, some of the genes identified with altered methylation in BA appear to include candidate genes for drug addiction, and other psychiatric disorders, such as intellectual disability, SCZ and ASD.

## Methods

### Subjects

This study was conducted in accordance with the Declaration of Helsinki and the Ethical Guidelines for Medical and Health Research Involving Human Subjects by the Japanese Ministry of Health, Labour and Welfare. All experimental procedures were approved by the Human Research Ethics Committee of Kyoto University Primate Research Institute, and the Ethics Committee of Kyowa Hospital. Upon enrolling in the study, written informed consent was obtained from all participants in advance of the experiments.

As a control (CT) group, blood samples were obtained from 24 healthy subjects without a history of smoking or psychiatric disorders (41.0 ± 7.43 years old; 9 males, 15 females). For the behavioral addiction (BA) group, blood samples were collected from hospitalized patients diagnosed with BA (n = 16; 36.9 ± 14.7 years old; 9 males, 7 females), which consisted of symptoms of gambling (n = 1), kleptomania (KM; n = 10) and paraphilia (PP; n = 5).

Blood sampling of approximately 3–5 mL from each participant was conducted in an EDTA-coated tube. Sampling in BA patients was conducted between 11:00 and 12:00, and in CT subjects between 16:00 and 17:00. Samples were frozen immediately after sampling and stored at − 30 °C in the freezer until processing for HPLC and DNA extraction.

### HPLC

High-performance liquid chromatography (HPLC) was conducted to investigate whole blood monoamine concentrations. For this assay, first, blood samples were centrifuged at 10,000 rpm for 5 min. Then, 490 μL from the surface of blood samples was placed into another 1.5 mL tube, and 10 μL of 10 pg/μL isoproterenol (ISO) as an internal standard was added to the samples, followed by 100 μL of 0.5 mol/L perchloric acid to remove all proteins. The samples were centrifuged again at 2,000 rpm for 15 min, and 10 μL each of supernatant from these samples was applied for HPLC.

Using a similar procedure described in our previous study^71^, HPLC was carried out using an HTEC-500 HPLC electrochemical detection system (Eicom, Tokyo, Japan) with the EICOMPAK SC-5ODS column and CA-ODS precolumn. The mobile phase consisting of 0.1 M acetic acid-citric acid buffer (pH 3.5) and methanol (83:17, v:v), along with 190 mg/L sodium 1-octanesulfonate and 5 mg/L EDTA-2Na, was used. The mobile phase flow rate was 500 μL/min, and the working electrode was set at + 750 mV against the Ag/AgCl reference electrode.

The standard with the known concentration of target substances (DA, 5-HT, NE, Epi, MHPG, NM, DOPAC, HVA, 3-MT, and 5-HIAA) and ISO were used to quantify and identify the peaks on the chromatographs. The quantification of target substances in a sample was based on the following formula: (*ISO*_*std*_*/TA*_*std*_) × (*TA*_*spl*_*/ISO*_*spl*_) × *A* × (*1/B*), where *ISO*_*std*_ and *TA*_*std*_ are the areas under the peaks of ISO and a target substance in the standard, whereas *ISO*_*spl*_ and *TA*_*spl*_ are the areas under the peaks of ISO and a target substance in a sample; *A* is the amount of ISO added to the sample; and *B* is the volume of the blood sample used.

### Genome-wide DNA methylation assay

Blood samples obtained from 10 CT, 5 KM, and 5 PP subjects were used for methylation assay. In CT and KM groups, 10 and 5 samples were selected from 24 CT and 10 KM subjects, respectively, based on characteristics of symptoms, for which the attending physician described as most typical, along with matching for age and sex.

Genomic DNA was extracted from whole blood samples with the ISOSPIN Blood and Plasma DNA Kit (Nippon Gene, cat. #312-08131) according to the protocol provided by the manufacturer. DNA quality was checked by the Picogreen (Promega, cat. #E2670) method using a synergy HTX Multi-Mode Reader, whereas the purity of DNA was assessed by NanoDrop spectrometry. In addition, DNA conditions were further assessed by gel electrophoresis. After these quality controls of extracted genomic DNA, 500 ng of DNA in each sample was bisulfite converted with the EZ DNA Methylation Kit (Zymo Research, cat. #D5001). Then, a genome-wide DNA methylation assay was conducted with an Illumina Infinium MethylationEPIC BeadChip kit (Illumina, cat. #WG-317-1001)^72^, and the array was scanned with an Illumina iScan scanner.

Array data were processed and analyzed using Illumina GenomeStudio v2011.1 (Methylatioin Module v1.9.0) and R 3.6.0 (https://www.r-project.org). Each methylation data point is represented by fluorescent signals from the M (methylated) and U (unmethylated) alleles. Background intensity computed from a set of negative controls was subtracted from each analytical data point. Thus, after image analysis and the extraction of raw data for 865,918 CpGs, preprocessing and quality checks were conducted to reduce systematic bias to avoid statistically erroneous conclusions through the correction, filtering, transformation, and normalization of data. Background correction and dye bias equalization were conducted with library (lumi) in R. Filtering was conducted for the detected CpGs by a detection p-value, excluding CpGs with detection p-value ≥ 0.05 in more than 25% of all samples, or p-value = NA in at least one sample. This filtering resulted in 845,978 CpGs to be analyzed. Then, beta mixture quantile (BMIQ) normalization^73^ was conducted to reduce assay bias using BMIQ in R, followed by data transformation to calculate β-value = (max(M, 0))/(|U| + |M| + 100), which is the ratio of fluorescent signal intensity of the methylated probe and the overall intensity (sum of methylated and unmethylated probe intensities). The β-value reflects the methylation level of each CpG site. A β-value of 0–1 was reported to signify the percentage of methylation, from 0 to 100%, respectively, for each CpG site. In addition, the M-value, which is the log2 ratio of the intensities of methylated probe versus unmethylated probe was also calculated for statistical analysis^74^. Using β- and M-values, the ∆mean (the difference between the average β-values fir the BA group and the CT group), odds ratio (by transforming ∆mean into M-value and measuring the ratio between unmethylated intensity and methylated intensity for the BA group versus that of the CT group), and fold change (the ratio of methylation rates between the BA group and the CT group) were also calculated for each CpG site.

### Data analysis

Comparisons between the BA group and the CT group and between the KM group and the PP group in HPLC and genome-wide methylation assays were conducted with unpaired *t* tests. A probability value of p < 0.05 was considered to indicate statistical significance.

Using the data of hyper- and hypomethylated CpG sites in BA patients compared to CT subjects from genome-wide methylation assays, gene network analysis was also conducted using the databases available on the internet. Whether hyper- and hypomethylated CpG sites in blood samples of BA patients were correlated with methylation status in brain tissues was analyzed with IMAGE-CpG (https://han-lab.org/methylation/default/imageCpG)^35^, gene network analysis was conducted with GeneMANIA (https://genemania.org/)^36^, tissue specific protein–protein interaction analysis and gene–disease association analysis were conducted with NetworkAnalyst (https://www.networkanalyst.ca/)^37^, and more detailed gene–disease analysis was conducted with DisGeNET (https://www.disgenet.org/)^38^, following the respective manuals at each website.

## Supplementary information

Supplementary Information 1.

Supplementary Information 2.

Supplementary Information 3.

Supplementary Information 4.

Supplementary Information 5.

Supplementary Information 6.

Supplementary Information 7.

Supplementary Information 8.

Supplementary Information 9.

## Data Availability

The datasets generated and analyzed during the current study are available as supplementary data. Additional data are available from the corresponding author upon reasonable request.
