# Supplementary material for: Monoamine and genome-wide DNA methylation investigation in behavioral addiction
Source: Sci Rep. 2020 Jul 16;10:11760. doi: 10.1038/s41598-020-68741-5 (PMC7366626; doi:10.1038/s41598-020-68741-5)
Supplement: Supplementary file 9 — Supplementary Information 9. [file 41598_2020_68741_MOESM9_ESM.pdf]

Supplemental Information

Monomaine and Genome-wide DNA Methylation Investigation in Behavioral Addiction

Y. Asaoka, M. J. Won, T. Morita, E. Ishikawa, Y.-A. Lee, & Y. Goto

Supplemental Figure S1
Reproducibility between samples

Supplemental Data S1
Hyper- and hypo-methylation sites in behavioral addiction patients compared to those in control subjects

Supplemental Data S2
Hyper- and hypomethylation sites in behavioral addiction that are correlated to those in brain tissues

Supplemental Data S3
Biological functions of genes with hyper- and hypomethylation in behavioral addiction

Supplemental Data S4
Tissue specific protein-protein interactions in the nucleus accumbens based on genes with hyper- and hypomethylation in behavioral addiction

Supplemental Data S5
Gene-disease associations based on the genes with hyper- and hypomethylation in behavioral addiction

Supplemental Data S6
Disease associations for genes with hyper- and hypomethylation in behavioral addiction

Supplemental Data S7
Hyper- and hypomethylation sites in patients with kleptomania compared to those in patients with paraphilia

Supplemental Data S8
Receiver operator characteristic analyses of DNA methylation.

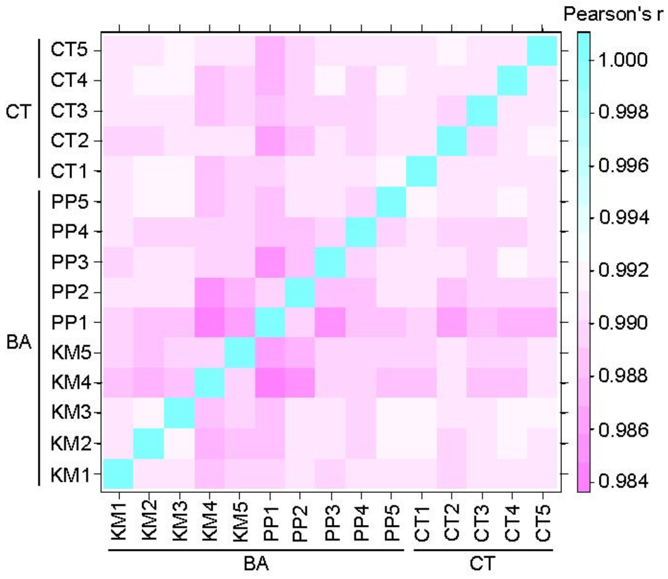

Supplemental Figure S1. Reproducibility between samples. A color-coded correlation matrix of Pearson's correlation coefficient (r) between samples with 1,000 randomly selected CpGs from each sample. BA: behavioral addiction, CT: control, KM: kleptomania, PP: paraphilia.
